# Unexpected Findings in a Child with Atypical Hemolytic Uremic Syndrome: An Example of How Genomics Is Changing the Clinical Diagnostic Paradigm

**DOI:** 10.3389/fped.2017.00113

**Published:** 2017-05-22

**Authors:** Eleanor G. Seaby, Rodney D. Gilbert, Gaia Andreoletti, Reuben J. Pengelly, Catherine Mercer, David Hunt, Sarah Ennis

**Affiliations:** ^1^Human Genetics and Genomic Medicine, Faculty of Medicine, University of Southampton, Southampton, UK; ^2^Wessex Regional Paediatric Nephro-Urology Service, Southampton Children’s Hospital, Southampton, UK; ^3^Faculty of Medicine, University of Southampton, Southampton, UK; ^4^Wessex Clinical Genetics Service, Princess Anne Hospital, Southampton, UK

**Keywords:** aHUS, genomics, JMML, preclinical cancer, whole-exome sequencing

## Abstract

*CBL* is a tumor suppressor gene on chromosome 11 encoding a multivalent adaptor protein with E3 ubiquitin ligase activity. Germline *CBL* mutations are dominant. Pathogenic *de novo* mutations result in a phenotype that overlaps Noonan syndrome (1). Some patients with *CBL* mutations go on to develop juvenile myelomonocytic leukemia (JMML), an aggressive malignancy that usually necessitates bone marrow transplantation. Using whole exome sequencing methods, we identified a known mutation in *CBL* in a 4-year-old Caucasian boy with atypical hemolytic uremic syndrome, moyamoya phenomenon, and dysmorphology consistent with a mild Noonan-like phenotype. Exome data revealed loss of heterozygosity across chromosome 11q consistent with JMML but in the absence of clinical leukemia. Our finding challenges conventional clinical diagnostics since we have identified a pathogenic variant in the *CBL* gene previously only ascertained in children presenting with leukemia. The increasing affordability of expansive sequencing is likely to increase the scope of clinical profiles observed for previously identified pathogenic variants and calls into question the interpretability and indications for clinical management.

## Case Presentation

Our patient presented aged 4 months with right-sided focal seizures. He had been born at term following an uncomplicated pregnancy. A brain magnetic resonance imaging (MRI) scan revealed a left cerebral artery infarct and occlusion of the left internal carotid artery with collateral flow in keeping with moyamoya phenomenon; sequelae have included a right hemiparesis and dysarthria.

At the age of 2 years, this patient had marked thrombocytopenia (platelets 26 × 10^9^/L, hemoglobin 115 g/L), mild proteinuria (urine protein/creatinine ratio 42 mg/mmol), and hypertension. Investigations revealed normal range renin, aldosterone, reticulocyte count, lactate dehydrogenase, von Willebrand factor, and ADAMTS13. He had a negative Coombs test, but low serum complement C3 (0.59 g/L, normal 0.75–1.65). Complement C4 was normal (0.14 g/L, normal 0.14–0.54). Alternative pathway hemolytic complement activity was low at 28% (normal 80–200%) but total hemolytic complement was normal at 92%, suggesting dysregulated activation of the alternative complement pathway. Red cell fragments were absent on blood film but the haptoglobin concentration was reduced at 0.17 g/L (normal 0.5–2.0). A karyotype was normal. Renal biopsy was unremarkable by light microscopy apart from light C3 staining along capillary walls. Electron microscopy confirmed endothelial cell separation from the glomerular basement membrane with accumulation of fluffy subendothelial material consistent with endothelial damage. Atypical hemolytic uremic syndrome was considered likely and possibly the cause of his cerebral infarct; he thus commenced eculizumab therapy. Sequencing of the coding regions and flanking sequences of *C3, CFI, CFB, CD46*, and *DGKE* revealed no pathogenic mutations. Sequencing of *CFH* revealed a heterozygous variant (c.G2850T:p.Q950H), which at the time was of unknown clinical significance.

**Figure 1:**
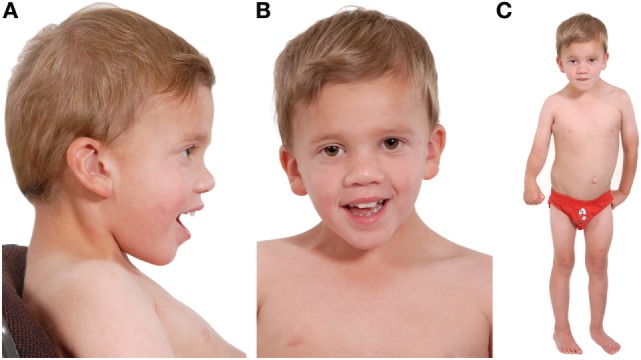
**Three photographs of our patient taken at the age of 4 years**. Photograph **(A)** shows a low posterior hairline with low-set posteriorly rotated ears, microcephaly, and mild frontal bossing. Photograph **(B)** shows down-slanting palpebral fissures and mild ptosis. He has dental crowding and a narrow, high-arched palate (not shown). Photograph **(C)** shows lasting damage from his cerebral infarct: the right upper limb is flexed and there is reduced muscle tone of the thigh in comparison to the left lower limb. His neck appears broad, with a broad thorax and wide-spaced nipples. His spleen measures 12 cm and marginally distorts the appearance of the abdomen. His skin is soft and mottled and he has clinodactyly. He is <0.4th centile for height, weight, and head circumference, and developmentally he is predicted to have skills appropriate for a 2½–3-year old. Noteworthy, during the antenatal period, marginal nuchal fold enlargement was documented but otherwise pregnancy and delivery were uneventful.

Examination at age 3 revealed dysmorphic features (Figure 1). His spleen was palpable 5 cm below the costal margin, and he had a right-sided hemiparesis with upper arm withdrawal reflex and down-going plantars. He had marked dysarthria and only spoke single words. Developmentally, he had skills appropriate for a 1½–2½-year old, which were attributed to his cerebral infarct. He had previously undergone orchidopexy to correct bilateral cryptorchidism. Despite an improvement in his platelet count following continued eculizumab therapy, he remained variably thrombocytopenic (platelet counts 85–181 × 10^9^/L) with marked splenomegaly. A bone marrow aspirate showed no impaired thrombocyte production or morphological abnormalities; therefore, his thrombocytopenia was attributed to hypersplenism.

The clinical hypothesis was an endothelial abnormality which interfered with complement regulation, possibly by reducing factor H binding, causing thrombotic microangiopathy (TMA) involving the kidneys and brain. The splenomegaly was not explained.

## Materials and Methods

WES was undertaken in an attempt to elucidate the pathophysiology. Genomic DNA was extracted from whole blood, and target capture was performed on Agilent’s SureSelect v5.0 (51 Mb). The enriched library was sequenced on the Illumina HiSeq2000. The identity and provenance of returned sequencing data were validated through application of an optimized genotyping panel (2). WES data were analyzed using an in-house pipeline as previously described (3, 4). Candidate genes were selected using curated databases of pathogenic variants associated with search terms applicable to the phenotype of interest.

## Results

In total, 24,955 variants were called with an average read depth of 58×. Of these, 470 variants were loss of function mutations, 2,631 were splicing variants, 11,146 were synonymous, and 10,708 were non-synonymous single nucleotide variants. Primary analysis comprised filtering on a targeted panel of 540 complement-associated genes; 916 variants were called. Variant prioritization identified two variants of unknown significance: the same variant in *CFH* (c.G2850T:p.Q950H) as found in the aHUS gene panel and a splicing variant in *CR1* (c.7252 + 1G>A). The results were equivocal.

**Figure 2:**
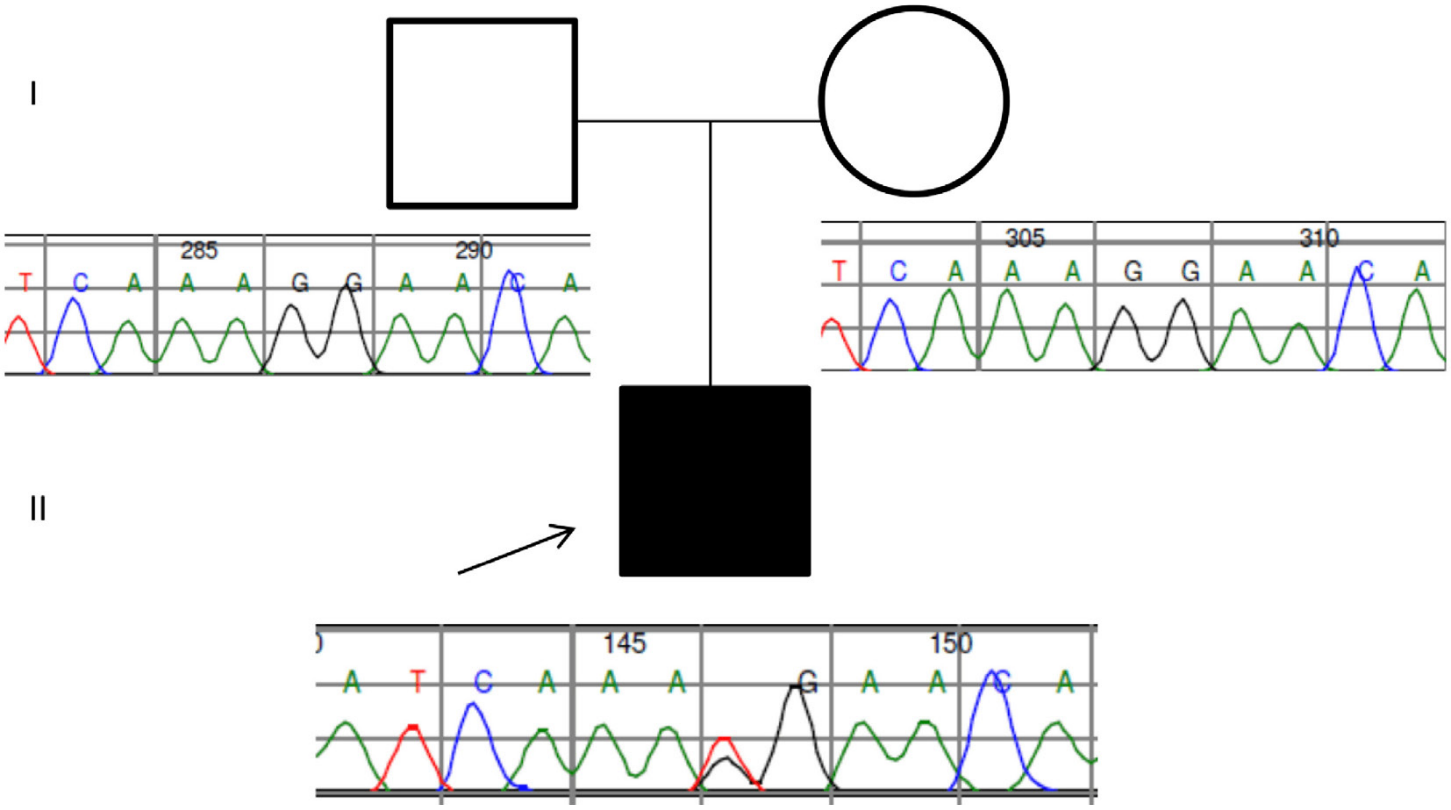
**Pedigree and Sanger traces of the patient/parent trio**. The proband (filled black square) has a *de novo CBL* heterozygous mutation of c.1096-1G>T, affecting the canonical splice site.

Two years later, this case was revisited following clinical review. Phenotypic information concerning moderate splenomegaly, persistent thrombocytopenia (despite eculizumab therapy, a normal bone marrow aspirate, and unchanged appearances of the cerebral magnetic resonance angiography) informed a revised analysis of further 44 genes collated from a literature search of PUBMED and the Human Gene Mutation Database using the search terms thrombocytopaenia, splenomegaly, and moyamoya. Filtering parameters reduced 51 variants to one heterozygous splicing variant in *CBL* (c.1096-1G>T) predicted to be pathogenic following application of the American College of Genetics and Genomics guidance (5). The mutation was validated by Sanger sequencing and segregation analysis confirmed *de novo* inheritance following the absence of the splicing mutation in both parents (Figure 2).

**Figure 3:**
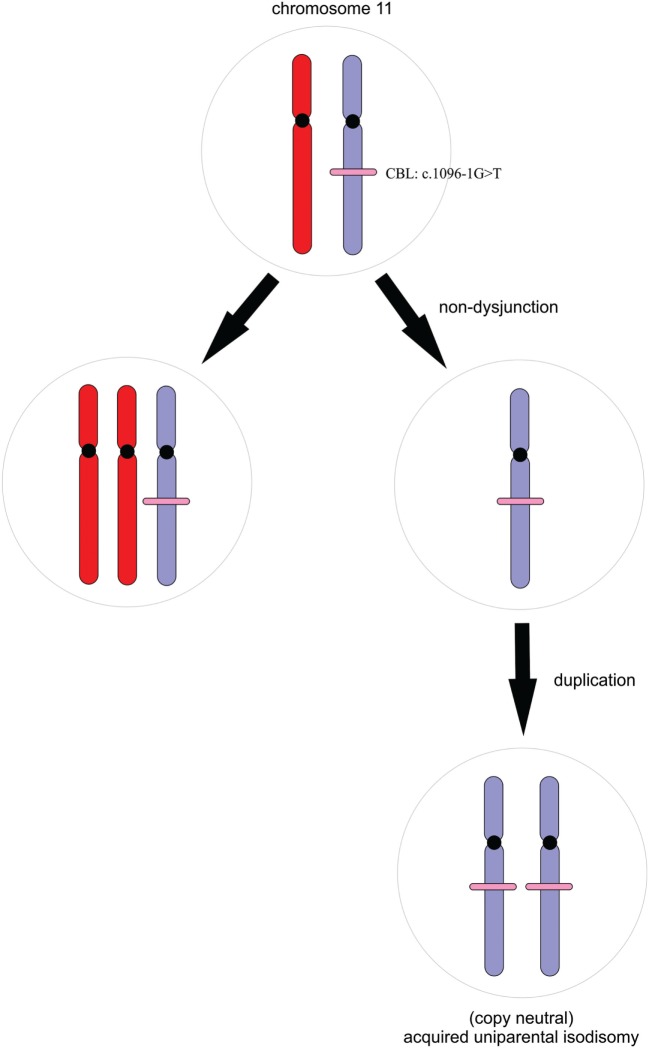
**A simple schematic illustrating the process of acquired uniparental isodisomy**. For simplicity, we illustrate complete chromosomal loss of heterozygosity. Non-dysjunction results in unequal chromosomal division during mitosis. Copy neutral loss of heterozygosity occurs with duplication of the mutant allele and loss of the wild-type without a change in copy number.

In children harboring germline *CBL* mutations, juvenile myelomonocytic leukemia (JMML) usually develops following somatic loss of heterozygosity (LOH) of chromosome 11q (6) although some patients develop JMML without LOH. In nearly all cases, the mutant allele is duplicated by acquired uniparental isodisomy, resulting in loss of the wild-type tumor suppressor allele and duplication of the oncogenic mutation (Figure 3) (1, 7, 8). WES data were, therefore, scrutinized for LOH across chromosome 11 in the region of the *CBL* locus; this was facilitated by peripheral blood-derived DNA. We retrospectively assessed LOH by plotting B-allele frequency ratios across the exome (Figure S1 in Supplementary Material) (9). On average, 70% of the sequenced reads mapping across chromosome 11q harbored the mutant allele compared with the reference. This significant allelic imbalance strongly suggested a clonal advantage of (a subset of) peripheral leukocytes consistent with myeloproliferation and a potential transformation to JMML (6, 7).

The two variants of unknown significance in *CR1* and *CFH* were also revisited. Sanger sequencing confirmed both heterozygous variants in the proband. Neither variant was *de novo*; the *CFH* variant was inherited from the proband’s asymptomatic father, and the *CR1* variant was inherited from his asymptomatic mother. To assess functional significance, red blood cells (RBCs), plasma and DNA were sent to the Jokiranta Research Group, at the University of Helsinki for functional analysis. *CR1* was ruled out as pathogenic following normal expression of the complement receptor 1 on erythrocytes with normal levels of C3dg on the RBC surface. The Q950H variant of Factor H is now known to impair factor H activity (10), providing a genetic explanation for the patient’s aHUS. As previously reported, most aHUS mutations show variable penetrance, providing explanation for why the proband’s father is unaffected (11).

## Discussion

Germline *CBL* mutations have been associated with Noonan-like syndrome, moyamoya, and vasculitis (12). Our patient displays many of the phenotypic features consistent with germline *CBL* mutations. Although his global developmental delay had been attributed to his cerebral infarct, review of his MRI scans suggests topographical inconsistencies; therefore, in retrospect, it is likely that a proportion of his developmental delay results from his *CBL* mutation.

Juvenile myelomonocytic leukemia is an aggressive, childhood myeloproliferative disease cured only by hematopoietic stem cell transplant (HSCT), yet hematological heterogeneity has been reported; some hematological abnormalities spontaneously resolve, while others progress aggressively (6, 13). Data analysis poses a difficult diagnostic dilemma; our patient is displaying evidence of myeloproliferation and subclinical JMML (by discovery of clonal expansion within the peripheral blood leukocyte population) despite not manifesting clinical leukemia; he has a normal peripheral blood film, white cell count, lactate dehydrogenase level, and no increase in peripheral blood monocytes. However, his monocytes were persistently elevated between 2011 and 2014, and he continues to have a borderline monocytosis. Furthermore, he has unremitting splenomegaly, a classical feature of JMML and has intermittent, mild anemia (hemoglobin concentrations 104–124 g/L) and thrombocytopenia. Interestingly, the proband’s thrombocytopenia did not fully resolve with eculizumab, potentially demonstrating marrow replacement by malignant cells or marrow failure; a common feature of JMML. This patient does not meet full hematological JMML diagnostic criteria, although he does meet oncogenetic diagnostic parameters (13) and would have met the diagnosis between 2011 and 2014 during which time he had persistent monocytosis. Even so, not all patients with *CBL* mutations and associated LOH will develop fulminant JMML that necessitates HSCT. CBL-associated JMML does not always follow an aggressive course and can spontaneously resolve without treatment (13). That said, in previous cases of *CBL* mutations involving the *same* splice site, the disease presented aggressively (6); indeed in one child, the mutation remained heterozygous in hematopoietic cells without evidence of LOH, yet the patient still required HSCT (14). Therefore, this finding poses a challenging clinical scenario, particularly since there is uncertainty regarding the disease trajectory.

**Figure 4:**
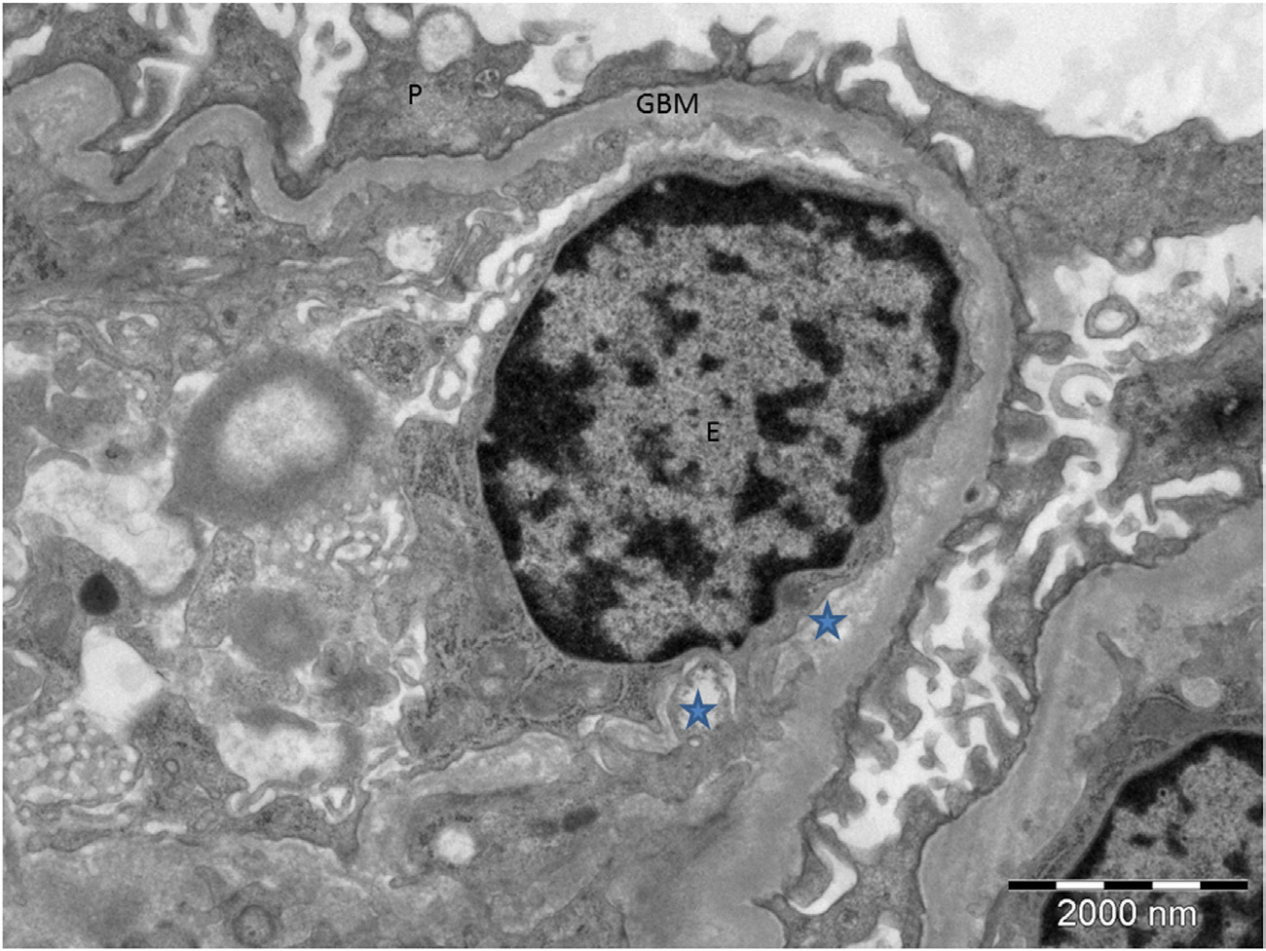
**Electron microscopy of the proband’s renal biopsy demonstrating tissue microangiopathy**. The stars show the subendothelial space, representing detachment of the endothelial cell from the basement membrane. P, podocyte; GBM, glomerular basement membrane; E, endothelial cell.

Two differential prognoses are as follows: (a) progression to an aggressive JMML if no potentially curative HSCT intervention is offered or (b) spontaneous regression (if not already regressed) of a relatively quiescent myeloproliferative cell population that appears aggressive in the literature due to ascertainment bias. Ultimately, the biggest challenge concerns the appropriate action(s) to take, especially since a decision to proceed to transplant is not without significant risk, and there is established phenotypic heterogeneity and variable expressivity among individuals with identical *CBL* mutations (15). The current recommendation for JMML secondary to *CBL* mutations is that of careful surveillance. Locatelli and Niemeyer recommend that in children harboring *CBL* mutations, the decision to proceed to transplantation should be carefully weighed. They recommend adopting a “watch and wait” approach with close follow-up to enable prompt diagnosis and subsequent action should the disease evolve (13). There is, however, speculation that HSCT may prevent further vascular complications, although this is yet to be definitively proved. HSCT recipients with JMML secondary to *CBL* mutations tend not to have further vasculitis, suggesting that these mutations cause endothelial damage (13). It seems likely, therefore, that abnormal endothelium combined with reduced complement factor H function may have “primed” the endothelium for complement mediated damage, resulting in TMA as shown on the renal biopsy (Figure 4). In view of the severity of his previous infarct and extensive changes in intracranial vessels on magnetic resonance angiography, there may be some justification in considering HSCT in this specific case, especially should a conservative approach result in further neurovascular or nephrological damage. However, this should be balanced against the risk of TMA following bone marrow transplantation in which the role of eculizumab is not well established.

This case also raises ethical questions with regard to return of information. There are concerns that informed consent for WES is insufficient in educating patients about the scope of potential results identified (16). When presented with data from an entire exome, there is always the possibility of incidentally discovering pathogenic mutations unrelated to the presenting phenotype (17). Although this was not an incidental finding *per se*, his pretest diagnosis was aHUS with atypical features. The exome analysis was primarily to elucidate a cause for his aHUS, since at the time of referral, the dysmorphology was not extensively documented and was presumed to be an unusual manifestation of TMA. His developmental delay had not been considered as independent to his infarct, and his only active clinical input was by the nephrology service. Revisiting exome data allowed for the discovery of pre-leukemia in addition to a monogenic explanation for his dysmorphology, moyamoya, and developmental delay. Relaying this information to his family necessitated reflection on how “informed” the consent truly was, as well as the magnitude, distress, and implications of finding “more than we bargained for.”

Our finding lies at the nexus of genomic and clinical, hematological, nephrological, and neurological diagnostics, necessitating a multidisciplinary convergence of genomic informaticians and clinicians in discussions concerning best practice. Since there is a relative dearth of evidence concerning children who harbor *CBL* mutations in the same splice site as our patient, and who do not progress to JMML, this paper attempts to highlight the variable expressivity of these mutations. We demonstrate how continued clinical review can inform a revised analysis of exome data and uncover unexpected findings that, in retrospect, highlight the limitations of biased and fallible clinical diagnostics. The ability to return to unbiased exome data without cost duplication is of huge diagnostic value, especially since there is no restriction to the number of times data can be revisited; for example, if the phenotype changed in the future or if functional studies reassigned a previously curated variant of unknown significance as a pathogenic allele. The full potential and utility of genomic data within the clinical setting are yet to be fully appreciated, but in the emerging genomics era, cases such as these will become increasingly prevalent, and the interpretation and translation of genomic data within clinical medicine has the potential to force a paradigm shift in clinical diagnostics with substantial prognostic impact.

## Author Contributions

ES: exomic data analysis, clinical data review, phenotyping, and initial draft manuscript. RG: clinical consultant, reviewed and revised the manuscript, approved the final manuscript, and supervision. GA: bioinformatics quality control analysis, exome data analysis, and approved the final manuscript. RP: exome data analysis, reviewed the manuscript, and approved the final manuscript. CM: clinical genetics consultant, documented phenotype, and approved the final manuscript. DH: genetics specialist trainee, documented phenotype, and approved the final manuscript. SE: exomic analysis oversight, reviewed and revised the manuscript, supervision, and approved the final manuscript.

## Conflict of Interest Statement

The authors declare that the research was conducted in the absence of any commercial or financial relationships that could be construed as a potential conflict of interest.

## Supplementary Material

Figure S1 **Segmented B allele frequency demonstrating significant loss of heterozygosity (LOH) on chromosome 11q**. The *y*-axis denotes the proportion of alternative **(B)** to reference **(A)** alleles across all called heterozygous variants. Heterozygous calls are expected to harbor 50% of the A allele and 50% of the B allele (one maternal and one paternal copy). A significant perturbance in this ratio (allelic imbalance) indicates LOH. Red boxes denote regions of LOH across chromosome 11q, and the green line shows segmented average of B allele frequency. Preliminary copy number variant analysis of chromosome 11 is consistent with (copy neutral) acquired uniparental isodisomy.
