# Supplementary figures and images for: Unexpected Findings in a Child with Atypical Hemolytic Uremic Syndrome: An Example of How Genomics Is Changing the Clinical Diagnostic Paradigm
Source: Front Pediatr. 2017 May 22;5:113. doi: 10.3389/fped.2017.00113 (PMC5438966; doi:10.3389/fped.2017.00113)

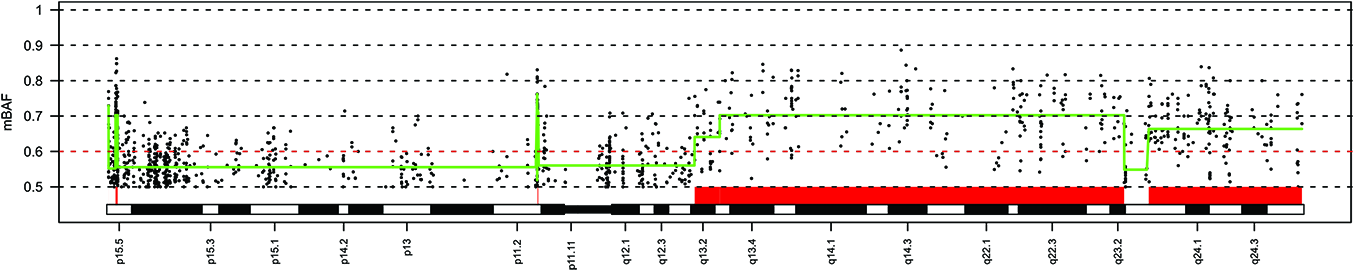

Supplement: Figure S1 — Segmented B allele frequency demonstrating significant loss of heterozygosity (LOH) on chromosome 11q. The y-axis denotes the proportion of alternative (B) to reference (A) alleles across all called heterozygous variants. Heterozygous calls are expected to harbor 50% of the A allele and 50% of the B allele (one maternal and one paternal copy). A significant perturbance in this ratio (allelic imbalance) indicates LOH. Red boxes denote regions of LOH across chromosome 11q, and the green line shows segmented average of B allele frequency. Preliminary copy number variant analysis of chromosome 11 is consistent with (copy neutral) acquired uniparental isodisomy. [file Image_1.TIF]
